# Early Onset Parkinson Syndrome, Type A Aortic Aneurysm and Noncompaction Associated With the Novel Variant c.2225C>T in MYH11: A Case Report

**DOI:** 10.7759/cureus.46793

**Published:** 2023-10-10

**Authors:** Josef Finsterer, Sounira Mehri

**Affiliations:** 1 Neurology, Neurology and Neurophysiology Center, Vienna, AUT; 2 Biochemistry Laboratory, LR12ES05, Nutrition-Functional Foods and Vascular Health, University of Monastir, Faculty of Medicine, Monastir, TUN

**Keywords:** aortic aneurysm, non-compaction, parkinson’s disease, cardiac involvement, myh11

## Abstract

Aortic aneurysm, left ventricular noncompaction, and early onset Parkinson syndrome have not been reported in association with *MYH11* variants. The patient is a 44-year-old male who developed a progressive ascending aortic aneurysm at age 30, requiring aortic repair at the age of 40. In addition, he developed Parkinson syndrome at the age of 37. He also suffered from myopia, hypothyroidism, arterial hypertension, hyperlipidemia, pre-diabetes, hyperbilirubinemia, obstructive sleep apnea syndrome (OSAS), and muscle cramps. Echocardiography and cardiac MRI showed left ventricular noncompaction. Genetic analysis revealed the novel heterozygous variant c.2225C>T (p.Ala742Val) in *MYH11*. Family history was positive for arterial hypertension (mother), carcinoma (brother), and diabetes (sister, father). There was consanguinity between the parents. With appropriate treatment, Parkinson syndrome and cardiac anomalies remained stable and there were no complications due to noncompaction or aortic repair. Considering that embryonic vascularisation may be involved in the pathophysiology of noncompaction and that *MYH11* is expressed in the myocardium, a causal relationship between the *MYH11* variant and noncompaction is conceivable.

In conclusion, this is the first case showing an aortic aneurysm associated with noncompaction and Parkinson syndrome in a carrier of the novel, heterozygous variant c.2225C>T in *MYH11*. Carriers of *MYH11* variants should be prospectively and systematically screened for multisystem diseases as soon as the genetic defect is discovered in order not to delay any necessary treatment. First-degree relatives should be screened for the *MYH11* variant of a family member to track the trait of inheritance and confirm its pathogenicity.

## Introduction

The *MYH11* gene encodes myosin heavy chain 11, which belongs to the myosin heavy chain family and is mainly expressed in smooth muscle cells [1]. The MYH11 protein is a subunit of a hexameric protein composed of two heavy chain subunits and two pairs of nonidentical light subunits [1]. MYH11 functions as an important contractile protein and converts chemical energy into mechanical energy through the hydrolysis of ATP [1]. Chromosomal rearrangements involving the *MYH11* gene are associated with acute myeloid leukaemia and sarcoma [2]. Mutations in *MYH11* are associated with multisystem disease, predominantly presenting as visceral myopathy (VM) [3], megacystis-microcolon-intestinal hypoperistalsis syndrome (MMIHS) [4,5], chronic intestinal pseudo-obstruction (CIPO) [6], and familial thoracic aortic aneurysm and dissection [7,8] but also with various other features [9-20]. Aortic aneurysm, noncompaction, also known as left ventricular hypertrabeculation (LVHT), and Parkinson syndrome have not been reported in association with *MYH11* variants.

## Case presentation

The patient is a 44-year-old male with Parkinson syndrome diagnosed at the age of 37, and type A aortic aneurysm with bicuspid aortic valve first diagnosed at age 30 (transverse diameter 46 mm). At age 40 the aortic aneurysm required aortic repair after having reached an aortic transverse width of 80 mm. He had initially received a D2-receptor agonist for Parkinson syndrome, which had to be discontinued after developing a shopping addiction and replaced with L-DOPA and the mono-amino-oxidase (MAO)-B inhibitor rasagiline. Medical history was also positive for myopia, hypothyroidism, arterial hypertension, hyperlipidemia, prediabetes, hyperbilirubinemia, obstructive sleep apnea syndrome (OSAS), chronic gastritis, folic acid deficiency, vitamin-D deficiency, depression with panic attacks, right supraspinatus tendon impingement, traumatic brain injury at the age of 39 with mild cerebral bleeding from a car accident, and severe SARS-CoV-2 infection complicated by respiratory insufficiency and questionable myocarditis. He also reported occasional double vision and muscle cramps in his right calf and fingers. Family history was positive for arterial hypertension (mother), carcinoma (brother), and diabetes (sister, father). There was consanguinity between the parents.

The clinical neurological examination at the age of 44 revealed a flat affect, depression, stiff neck muscles, hypomimia, myopia, dysosmia, hypesthesia of digits 1-3 of the right upper extremity, resting tremor with right-sided predominance, which increased in posture and intention, mild right-sided ataxia, but pronounced static ataxia with tendency to fall. There was no cognitive impairment, hypoacusis or pupillary dysmotility.

**Figure 1:**
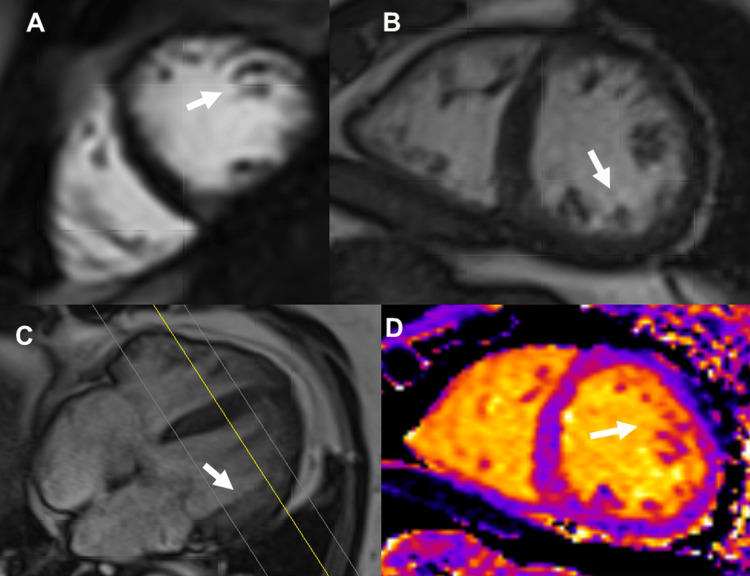
Cardiac MRI images Cardiac MRI showing extensive hypertrabeculation of the left ventricular myocardium distally to the papillary muscles. Panel A: Short-axis view (DYN_ sBTFE_5sl); Panel B: Short-axis view (Trufi Cine SAX); Panel C: Long-axis view (Trufi Cine 4CH); Panel D: Axial view (T1 Map SAX MOCO T1). After the application of the contrast medium, no LGE could be documented.

Blood tests only showed a hemoglobin A1C (HbA1c) value of 6.1 (n, <5.7) and a bilirubin value of 2.27 mg/dl (n, 0.3-1.2 mg/dl). Cerebral MRI revealed a small hemosiderin deposit in the right temporal region, a pineal cyst, and hypoplasia of the right vertebral artery, which was confirmed by carotid ultrasound. Nerve conduction studies of the right median and ulnar nerves revealed neither carpal tunnel syndrome nor ulnar sulcus syndrome. Transthoracic echocardiography revealed dilatation of the ascending aorta to 49 mm, focal aortic valve sclerosis, and hypertrabeculation, but was otherwise normal. Holter monitoring revealed no evidence of malignant ventricular arrhythmias (MVAs). Cardiac MRI showed left ventricular hypertrabeculation but normal cavity and wall dimensions and normal systolic function (Figure 1). There was no late gadolinium enhancement (LGE). Genetic testing of suspected arrhythmogenic right ventricular cardiomyopathy (ARVC) using a panel revealed the heterozygous variant c.2225C>T (p.Ala742Val) in *MYH11*. A panel test on hereditary Parkinson syndrome (GBA, LRRK2, PARK2, PARK7, PINK1, SNCA, VPS35) was nonconclusive.

His most recent medications included acetylsalicylic acid, atorvastatin, L-thyroxin, L-DOPA, rasagiline, amlodipine, candesartan, bisoprolol, and beclomethasone/formoterol.

## Discussion

The patient is of interest for a novel heterozygous variant of MYH11, which manifested phenotypically as aortic aneurysm, Parkinson syndrome, and noncompaction. Whether the detected heterozygous *MYH11* variant was actually responsible for the clinical manifestations remains speculative, but several previous studies reported that heterozygous *MYH11* variants can be pathogenic (Table 1) [3-20]. Except for aortic aneurysm [7,8], the other clinical manifestations of the index patient were not reported in association with *MYH11* variants. A causal relationship between the *MYH11* variant c.2225C>T and arterial hypertension and hyperlipidemia is unlikely, as they have not been reported in association with other *MYH11* variants. However, since *MYH11* is expressed in cardiomyocytes and neurons, it is conceivable that LVHT and Parkinson syndrome are causally linked.

**Table 1:** Patients carrying MYH11 variants (not including BFbeta/MYH11 fusion genes in leukaemia and sarcoma) and reported during the last 10 years until the end of August 2023 AHT: arterial hypertension, CAKUT: congenital anomalies of the kidney and urinary tract, chet: compound heterozygote, CIPO: chronic, intestinal pseudoobstruction, het: heterozygous, hom: homozygous, MMIHS: megacystis microcolon intestinal hypoperestalsis syndrome, nr: not reported, PBS: prune belly syndrome, VM: visceral myopathy, y: years

Age (y)	Sex	Variant	Dosage	Phenotype	Reference
Cardiovascular / cerebrovascular manifestation					
25	f	c.3728T>C	het	patent ductus, aortic dissection	Pan et al. 2023 [10]
12	f	IVS32G>A	nr	steno-occlusive arteriopathy with stroke	Raghuram et al. 2023 [11]
0	m	c.1502G>A	het	fetal hydrops, aortic tortuosity, fractures, hernia	Bharadwaj et al. 2022 [12]
22	m	c.4578+3A>C	het	aortic dissection, aortic aneurysm	Chesneau et al. 2021 [13]
45	f	c.4578+3A>C	het	aortic dissection, vertebrostenosis, pneumothorax	Chesneau et al. 2021 [13]
57	f	nr	nr	aortic aneurysm, dissection, Loeys-Dietz syndrome	Yoneyama et al. 2021 [14]
29	f	c.5373G>A	het	cerebral artery stenosis	Larson et al. 2020 [15]
0	nr	c.4366A>C	het	ductus arteriosus aneurysm	Ardhanari et al. 2020 [16]
72	nr	c.4658C>T	het	aortic aneurysm	Pucci et al. 2020 [17]
41	f	c.4658C>T	het	aortic aneurysm	Pucci et al. 2020 [17]
32	m	c.2156G>A	nr	aortic dissection	Yamasaki et la. 2019 [7]
2	f	c.4404G>A	het	Moya-Moya-like cerebrovascular disease	Keylock et al. 2018. [18]
0.5	f	c.5273G>A	het	middle cerebral artery aneurysm	Ravindra et al. 2016 [19]
40	m	c.3791T>C	nr	aortic aneurysm / dissection, patent ductus arteriosus	Takeda et al. 2015 [8]
13	m	nr	nr	aortic aneurysm, iris flocculi, miosis	Risma et al. 2014 [20]
1	f	c.2076C>T	nr	coarctation, arterial tortuosity	Loup et al. 2013 [21]
Gastrointestinal and urogenital manifestations					
1.5	nr	c.2809_2810del	chet	pulmonary, MMIHS, mydriasis, hypoacusis	Yetman et al. 2018 [5]
nr	m	c.3766A>C	nr	PBS, hydronephrosis	Geraghty et al. 2023 [22]
nr	m	c.5819del	nr	CAKUT, hydronephrosis	Geraghty et al. 2023 [22]
nr	f	c.5819del	nr	CIPO, myopathy	Geraghty et al. 2023 [22]
nr	m	c.5819del	nr	CIPO, myopathy	Geraghty et al. 2023 [22]
nr	m	c.5819del	nr	PBS, urinary retention	Geraghty et al. 2023 [22]
nr	m	c.3421A>C	nr	AHT, renal insufficiency	Geraghty et al. 2023 [22]
43	m	c.5819delC	het	CIPO, VM	Li et al. 2022 [3]
8	nr	p.G714X	chet	VM	Kapur et al. 2023 [23]
20	nr	16p13.11del	nr	VM	Kapur et al. 2023 [23]
nr	nr	nr	nr	MMIHS	Prathapan et al. 2021 [24]
nr	nr	c.5819_5820insCA	het	Gastro-intestinal dysmotility, hernia, stricture	Gilbert et al. 2020 [25]
nr	nr	c.5819el	het	Gastro-intestinal dysmotility syndrome	Gilbert et al. 2020 [25]
7	f	c.379C>T	chet	MMIHS	Kloth et al. 2019 [26]
nr	m	c.3598A>T	hom	MMIHS	Gauthier et al. 2015 [27]
29	f	c.2051G>A	chet	MMIHS	Wang et al. 2019 [4]
63	m	c.5819delC	het	CIPO, megacystis	Dong et al. 2019 [6]
70	m	c.5819delC	het	CIPO, megacystis,	Dong et al. 2019 [6]
66	m	c.5819delC	het	CIPO, megacystis, bowel obstruction	Dong et al. 2019 [6]
22	m	c.5819delC	het	CIPO, megacystis	Dong et al. 2019 [6]
40	f	c.5819delC	het	CIPO, rectal prolapse	Dong et al. 2019 [6]
53	m	c.5819delC	het	CIPO, megacystis	Dong et al. 2019 [6]
58	m	c.5819delC	het	CIPO, malrotation	Dong et al. 2019 [6]

The patient reported no gastrointestinal symptoms suggestive of VM, CIPO, or MMIHS, nor any recurrent fractures, visual disturbances, hypoacusis, stroke, urinary problems, or renal disease, as previously reported in *MYH11* mutation carriers (Table 1) [9-20]. Whether Parkinson syndrome was due to cerebrovascular involvement or another cause remains controversial. Whether the hyperbilirubinemia was due to the involvement of the biliary system has not been investigated.

LVHT is a morphologic abnormality of the left ventricular myocardium that is usually congenital and rarely acquired [9]. It is associated with various genetic defects and chromosomal aberrations, but a causal relationship between all of these genetic defects and noncompaction has not yet been proven. The diagnosis of noncompaction is usually made using echocardiography or cardiac MRI according to different criteria if there is an increased number of trabeculae distal to the papillary muscles. In some cases, LGE can be documented when a contrast medium is applied. Noncompaction can be complicated by cardioembolism due to the formation of thrombi in the intertrabecular spaces, heart failure, and MVAs with sudden cardiac death. None of these possible complications were noted in the index patient. However, one argument for causality is that, in addition to aortic dissection, other cardiac anomalies such as patent ductus arteriosus [11] or bicuspid aortic valves (index case) have also been reported in *MYH11* mutation carriers (Table 1) [13].

The cause of double vision and muscle cramps remains speculative, but there are various speculations to explain these symptoms. Skeletal muscle myopathy has previously been reported in carriers of MYH11 variants. Involvement of the cerebral arteries or the extremity arteries is also conceivable. However, the fact that no previous or acute stroke was detected on cerebral MRI and arterial pulses were easily palpable on clinical examination and his carotid ultrasound was normal argues against cerebrovascular or peripheral artery involvement. One argument against myopathy in the index case is that creatine kinase levels were within the normal range on every measurement.

A limitation of the study is that other family members were not screened for aortic aneurysm or noncompaction, the index patient’s parents were not screened for the *MYH11* variant, and the index patient was not tested for chromosomal abnormalities. Detection of the index patient’s *MYH11* variant in first-degree relatives could provide a strong argument for causality between the variant and the phenotype. It would also have a strong impact on genetic counselling, particularly for relatives who wish to have children.

## Conclusions

This case is the first to suggest that the novel, heterozygous variant c.2225C>T in *MYH11* can manifest phenotypically not only with aortic aneurysm as previously reported but also with noncompaction and Parkinson syndrome. However, a causal connection between the novel *MYH11* variant and heart and brain diseases still needs to be clarified. The case also supports the notion that both homozygous and heterozygous *MYH11* variants can be pathogenic and cause multisystem diseases, primarily affecting the arteries of the heart and brain, the urinary tract, the biliary system, and the gastrointestinal tract. Future studies should focus on a possible causal relationship between the novel *MYH11* variant and myocardial abnormalities, as well as between the *MYH11* variant and cerebral disease, including Parkinson syndrome. Future studies should also examine the pathogenicity of the c.2225C>T variant. Carriers of MYH11 variants should be prospectively and systematically screened for multisystem diseases as soon as the genetic defect is discovered in order not to delay necessary treatment. First-degree relatives should be screened for the *MYH11* variant of a family member to track the trait of inheritance and confirm its pathogenicity.
